# Regular snoring is associated with uncontrolled hypertension

**DOI:** 10.1038/s41746-024-01026-7

**Published:** 2024-02-17

**Authors:** Bastien Lechat, Ganesh Naik, Sarah Appleton, Jack Manners, Hannah Scott, Duc Phuc Nguyen, Pierre Escourrou, Robert Adams, Peter Catcheside, Danny J. Eckert

**Affiliations:** 1 Adelaide Institute for Sleep Health and FHMRI Sleep Health, College of Medicine and Public Health, Flinders University, Adelaide, Australia; 2 Centre Interdisciplinaire du Sommeil, Paris, France

**Keywords:** Epidemiology, Respiratory tract diseases, Hypertension

## Abstract

Snoring may be a risk factor for cardiovascular disease independent of other co-morbidities. However, most prior studies have relied on subjective, self-report, snoring evaluation. This study assessed snoring prevalence objectively over multiple months using in-home monitoring technology, and its association with hypertension prevalence. In this study, 12,287 participants were monitored nightly for approximately six months using under-the-mattress sensor technology to estimate the average percentage of sleep time spent snoring per night and the estimated apnea-hypopnea index (eAHI). Blood pressure cuff measurements from multiple daytime assessments were averaged to define uncontrolled hypertension based on mean systolic blood pressure≥140 mmHg and/or a mean diastolic blood pressure ≥90 mmHg. Associations between snoring and uncontrolled hypertension were examined using logistic regressions controlled for age, body mass index, sex, and eAHI. Participants were middle-aged (mean ± SD; 50 ± 12 y) and most were male (88%). There were 2467 cases (20%) with uncontrolled hypertension. Approximately 29, 14 and 7% of the study population snored for an average of >10, 20, and 30% per night, respectively. A higher proportion of time spent snoring (75th vs. 5th; 12% vs. 0.04%) was associated with a ~1.9-fold increase (OR [95%CI]; 1.87 [1.63, 2.15]) in uncontrolled hypertension independent of sleep apnea. Multi-night objective snoring assessments and repeat daytime blood pressure recordings in a large global consumer sample, indicate that snoring is common and positively associated with hypertension. These findings highlight the potential clinical utility of simple, objective, and noninvasive methods to detect snoring and its potential adverse health consequences.

## Introduction

Snoring is common and reflects narrowing and tissue vibration of upper airway soft tissues and structures^1^. The prevalence of habitual loud snoring in the general population remains unclear and is challenging to define in the absence of standardized approaches for snoring assessments, knowledge regarding clinical impacts of snoring itself, and reliance, in most existing epidemiological studies, on self- or bed partner-reports of snoring frequency and/or intensity. For example, a meta-regression of 63 studies reported a snoring prevalence between 2 and 83% in men and between 1 and 71% in women^2^. Snoring prevalence in clinical populations is also unclear. Snoring is a major feature of sleep-disordered breathing, including hypopnea events, which reflect partial airway obstruction typically with snoring. Consequently, most, but not all, individuals with obstructive sleep apnea (OSA) frequently snore loudly on most nights. OSA severity is usually categorized using clinical cut-offs^3^ (<5 events/h sleep = no OSA, $\ge$5 and <15 = mild, $\ge$15 and <30 = moderate and $\ge$30 events/h sleep = severe OSA); and more severe OSA is typically associated with more snoring during the night^4^. However, based on self-report snoring assessments in one of the largest epidemiological studies of sleep-disordered breathing effects on health, the Sleep Heart Health Study (SHHS), one-third of participants with OSA did not report any snoring while one-third of participants who were snorers did not meet criteria for OSA^5^. Thus, self-reported snoring assessments may not be sufficiently reliable to evaluate potential snoring impacts on cardiovascular health outcomes.

Physiological consequences of partial upper airway obstruction and snoring include substantial negative intrathoracic pressure swings that increase transmural pressure pre- and after-loads on the heart and blood pressure surges associated with brief awakenings from sleep (neuro-cortical arousals). Tissue vibrations may also injure the upper airway and surrounding tissues, including the carotid arteries^6,7^.

Snoring has been associated with multiple sub-clinical markers of cardiovascular pathology, including elevated blood pressure^8–10^, increased carotid-intima-media thickness^11^, stenosis^12^, and atherosclerosis^13^. These effects could partly reflect mechanical stress imposed by snoring vibrations on the upper airway in combination with a range of shared risk factors for OSA and cardiovascular disease, such as obesity and a sedentary lifestyle. In addition, snoring sound pressure levels are often substantially higher than indoor nighttime noise levels recommended by the World Health Organization^14^, and comparable to levels associated with poor cardiovascular health, including high blood pressure^14,15^. Thus, loud snoring may interfere with recuperative sleep and contribute to hypertension risk and other adverse outcomes in snorers and their bed partners through noise disturbance effects. Snoring could, therefore, be an important risk factor for hypertension and incident cardiovascular events, as recently shown with incident stroke in the Sleep Apnea Cardiovascular Endpoints (SAVE) trial^16^. However, in other studies, snoring was not associated with all-cause mortality or incident cardiovascular event or stroke^4,17^. Thus, the current evidence is conflicting, which may reflect the shortcomings of prior attempts to quantify snoring and the need for systematic and objective assessment of snoring.

Furthermore, large night-to-night variability in markers of OSA severity, such as the apnea-hypopnea-index, is now well established^18–21^. While no studies have examined night-to-night variability in objective snoring assessments, given the mechanistic overlap between hypopneic events and snoring, single-night snoring assessments may also not reliably reflect typical snoring and cumulative exposure risks over time.

Therefore, the existing evidence for associations between snoring and cardiovascular health outcomes remains limited and is primarily based on self-reported snoring assessments or clinical/epidemiological samples with single-night objective snoring assessments in relatively small sample sizes. This study, therefore, aimed to determine the prevalence of snoring and its association with hypertension prevalence using multi-night objective assessment of snoring and multiple daytime blood pressure assessments in a large sample population.

## Results

### Participant characteristics

The characteristics of the 12,287 participants included in this study are summarized in Table 1. Participants were middle-aged (~50 years), generally overweight (BMI ~ 28 kg/m^2^) and predominantly male (88%). Each participant had a median [IQR] of 29 [12, 81] repeat blood pressure recordings. Each participant also had an average of 181 ± 69 (mean ± SD) replicate recordings of sleep and snoring throughout the study period. The vast majority of sleep and snoring data were acquired from consecutive nights (~6 nights per week on average, see Table 1). Approximately 45, 29, 14, and 7% of the study population snored for more than 5, 10, 20, and 30% of the night, respectively. For men, these rates were 46, 30, 15, and 8%, whereas for women the equivalent rates were 36, 22, 9, and 4%.

**Table 1:** Baseline and snoring duration characteristics of the study population

			Quartile of snoring duration (% of total sleep time)			
		Overall	Q1 0 to 0.9%	Q2 0.9 to 4%	Q3 4 to 12%	Q4 12 to 83%
**n**		12,887	3072	3072	3071	3072
**Age, years**		50 (12)	46 (13)	49 (12)	51 (11)	52 (11)
**BMI, kg.m**^**-2**^		28 (6)	27 (5)	27 (5)	29 (5)	31 (6)
**Sex, n (%)**	**males**	10,868 (88%),	2611 (85%)	2696 (88%)	2758 (90%)	2803 (91%)
	**females**	1419 (12%)	461 (15%)	376 (12%)	313 (10%)	269 (9%)
**Hypertension,** ***n*** **(%)**	**no**	9820 (80%)	2675 (87%)	2551 (83%)	2387 (78%)	2037 (66%)
	**yes**	2467 (20%)	397 (13%)	521 (17%)	684 (22%)	1035 (34%)
**Systolic blood pressure** ≥ **140** **mmHg**		1537 (13%)	229 (7%)	302 (10%)	388 (13%)	618 (20%)
**Diastolic blood pressure** ≥ **90** **mmHg**		1855 (15%)	247 (8%)	367 (12%)	493 (16%)	748 (24%)
**Mean systolic blood pressure, mmHg**		127 (12)	123 (12)	125 (11)	127 (11)	131 (12)
**Mean diastolic blood pressure, mmHg**		82 (8)	79 (8)	81 (8)	83 (8)	85 (8)
**Average AHI, events/h**		12.5 (13.9)	7.5 (10.0)	9.4 (11.0)	12.1 (12.2)	21.1 (17.2)
**Snoring duration, %**		8.9 (11.9)	0.3 (0.3)	2.2 (0.9)	7.4 (2.3)	25.7 (12.6)
**Average number of nights**		181 (69)	179 (70)	183 (68)	181 (68)	180 (68)
**Average number of nights per week**		6 (1)	6 (1)	6 (1)	6 (1)	6 (1)

The Spearman correlation coefficient between snoring duration and mean eAHI was 0.42 (*p* < 0.001). Participants with moderate and severe OSA exhibited substantially higher proportions of sleep time with snoring (Table 2) compared to participants with mild OSA, who were quite evenly represented in each snoring quartile. Only 9% of participants with severe OSA did not snore (lowest quartile of snoring). Participants without OSA had the lowest proportion of sleep time with snoring.

**Table 2:** OSA severity [n (%)] in relation to snoring duration quartiles

		Quartiles of percent of sleep time spent snoring			
		0 to 0.9%	0.9 to 4%	4 to 12%	12 to 83%
**Obstructive sleep apnea severity category**^a^	**No OSA**	1727 (38%)	1413 (31%)	974 (22%)	405 (9%)
	**Mild**	920 (22%)	1070 (25%)	1250 (29%)	996 (24%)
	**Moderate**	303 (13%)	420 (19%)	596 (26%)	943 (42%)
	**Severe**	115 (9%)	171 (13%)	254 (20%)	730 (58%)

^a^OSA category was defined using standard clinical cut-offs (<5 events/h sleep = no OSA, ≥5 and <15 = mild, ≥15 and <30 = moderate and ≥30 events/h sleep = severe OSA).

### Snoring and blood pressure

**Fig. 1:**
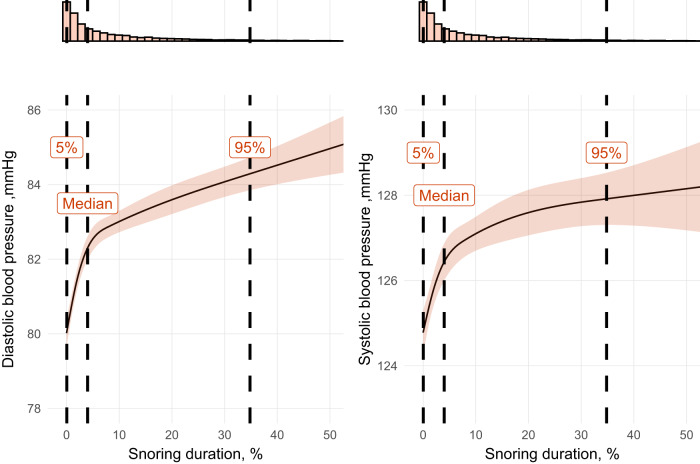
Associations between snoring duration with systolic and diastolic blood pressure. Models use 3 knots restricted cubic spline for snoring duration.

Increased snoring duration was associated with both increased systolic and diastolic blood pressure, even after adjusting for averaged eAHI, age, BMI, and sex (Fig. 1). The association between snoring duration and blood pressure modelled without interactions is depicted in Fig. 1. These data show ~3 and ~4 mmHg increases in systolic and diastolic blood pressure for frequent regular snoring versus infrequent snoring independent of age, BMI, sex, and mean eAHI, respectively. While there were significant interactions between obese and nonobese, ≤50 years old vs. >50 years old (median age) in the association between snoring duration and systolic and diastolic blood pressure, the effect size of the interaction was relatively small, with only a ~1 to 2 mmHg difference between BMI and age categories (Supplementary Fig. 1 and Supplementary Table 1).

Similar results were observed when OSA severity was categorized, and snoring duration was divided into quartiles (Table 3). In this model, severe OSA with no snoring was associated with 3.6 mmHg and 3.5 mmHg higher systolic and diastolic blood pressure compared to no snoring or OSA. Furthermore, participants with no OSA but high snoring (quartile 4) had a 3.8 mmHg and 4.5 mmHg higher systolic and diastolic blood pressure compared to participants with no sleep apnea or no snoring. Hence, the association between severe OSA-alone and blood pressure had a similar effect size to the association between snoring-alone and blood pressure.

**Table 3:** Associations between blood pressure with OSA severity categories and snoring duration

		Quartiles of snoring duration			
		0 to 0.9%	0.9 to 4%	4 to 12%	12 to 83%
**Systolic blood pressure**					
**Obstructive sleep apnea severity category**	**No OSA**	0 (ref)	1.2 (0.4,1.9)	2.4 (1.5, 3.2)	3.8 (2.6, 4.9)
	**Mild**	1.1 (0.3, 2.0)	2.1 (1.3, 3.0)	2.9 (2.1, 3.7)	3.6 (2.7, 4.4)
	**Moderate**	2.6 (1.3, 3.9)	2.5 (1.3, 3.6)	3.0 (1.9, 4.0)	4.3 (3.4, 5.2)
	**Severe**	3.6 (1.6, 5.7)	4.2 (2.5, 5.9)	3.9 (2.5, 5.4)	5.5 (4.5, 6.5)
**Diastolic blood pressure**					
**Obstructive sleep apnea severity category**	**No OSA**	0 (ref)	1.9 (1.4, 2.5)	2.9 (2.3, 3.5)	4.5 (3.6, 5.3)
	**Mild**	1.6 (1.0, 2.2)	3.1 (2.5, 3.7)	4.3 (3.7, 4.9)	5.0 (4.4, 5.6)
	**Moderate**	3.2 (2.3, 4.2)	3.0 (2.2, 3.9)	4.0 (3.3, 4.8)	5.4 (4.8, 6.1)
	**Severe**	3.5 (2.1, 5.0)	3.1 (1.9, 4.3)	4.5 (3.4, 5.5)	6.3 (5.6, 7.0)

Displayed values are β (95% CI) of the association between an OSA/snoring group with the reference group (no-OSA and lowest snoring quartile). Models were adjusted for sex, age and BMI. OSA severity categories were defined using standard clinical cut-offs of the mean apnoea-hypopnoea index (<5 = no OSA, ≥ 5 and < 15 = mild, ≥ 15 and < 30 = moderate, and ≥ 30 events/h sleep= severe OSA).

### Snoring and uncontrolled hypertension

**Fig. 2:**
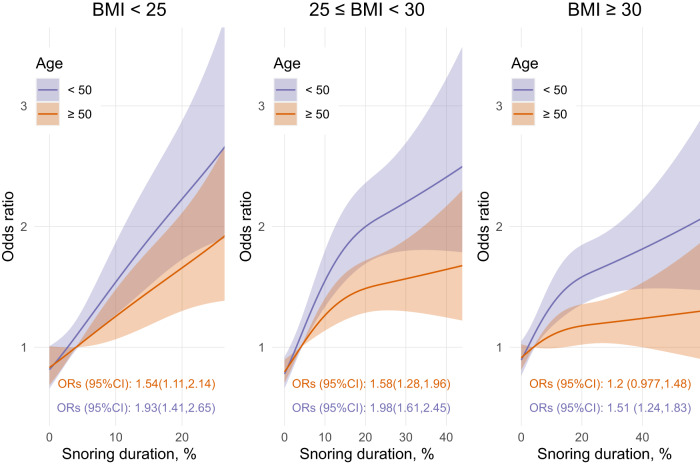
Associations between snoring duration with hypertension. Models use 3 knots restricted cubic spline and interaction with age categories (median split in years) and BMI categories (kg/m^2^). ORs (95%CI) represents the difference between the 5% and the 75% percent of the snoring duration distribution. Note that the 5% and 75% percent were determined separately for each BMI category; hence, slightly different x-axis.

Snoring duration was significantly associated with uncontrolled hypertension. The interaction between snoring duration and sex was not significant (*p* = 0.34). However, there were significant interactions between snoring and age (*p* = 0.003) and snoring and BMI (*p* < 0.001), with stronger associations in participants ≤ 50 years (Fig. 2) compared to those > 50 years and in participants with a BMI < 30 compared to ≥ 30 kg/m². In all instances, snoring was significantly associated with uncontrolled hypertension, from a 20% increased likelihood of uncontrolled hypertension in those aged >50 years and obese to a 98% increase in those aged ≤ 50 years with body weight in the normal BMI range.

Without considering the interactions, snoring duration was associated with an 87% increase in uncontrolled hypertension likelihood (75^th^ vs. 5^th^ percentile; 12% vs. 0.04%; OR [95%CI]; 1.87 [1.63, 2.15]). The association was nonlinear and is shown in Supplementary Fig. 2, with a reference point using the median snoring duration.

Similar results were also observed when OSA severity was categorized, and snoring duration was divided into quartiles (Table 4). The association between severe OSA-alone and uncontrolled hypertension showed a similar effect size to the association between the highest snoring duration-alone and uncontrolled hypertension (2.56-fold versus 2.73-fold increase).

**Table 4:** Associations between hypertension likelihood with OSA severity categories and quartile of % of sleep time spent snoring

		Quartiles of percent of sleep time spent snoring			
		0 to 0.9%	0.9 to 4%	4 to 12%	12 to 83%
**Obstructive sleep apnea severity category**	**No OSA**	1 (ref)	1.47 (1.17, 1.86)	1.81 (1.42, 2.30)	2.73 (2.05, 3.64)
	**Mild**	1.50 (1.16, 1.94)	1.86 (1.47, 2.37)	2.32 (1.86, 2.90)	2.86 (2.28, 3.58)
	**Moderate**	1.57 (1.09, 2.27)	2.02 (1.49, 2.76)	2.32 (1.77, 3.03)	3.08 (2.45, 3.89)
	**Severe**	2.56 (1.59, 4.14)	3.04 (2.04, 4.52)	2.83 (2.01, 3.97)	3.94 (3.09, 5.03)

Displayed values are ORs (95% CI) of the association between an OSA/snoring group with the reference group (no-OSA and lowest snoring quartile). Models were adjusted for sex, age and BMI.

### Primary snoring and uncontrolled hypertension

To test for the potential effects of primary snoring on uncontrolled hypertension, a similar analysis was applied to all participants with eAHI <5 events/h. After excluding participants with an eAHI ≥ 5 events/h, 4,529 (37%) participants remained, within which there were 607 (13.4%) cases of uncontrolled hypertension. The association between snoring duration and uncontrolled hypertension remained significant in this group. Participants who snored 5% (75^th^) of the night vs. those who did not (5^th^ percentiles; < 0.1%), had an 89% higher prevalence of uncontrolled hypertension (OR [95%CI], 1.89 [1.44, 2.46]), independent of age, sex, and BMI. The association between snoring and uncontrolled hypertension was nonlinear, as shown in Supplementary Fig. 3.

### Sensitivity analyses

Further adjusting for total sleep time did not change the main findings, and snoring duration in this model was associated with an 88% increase in uncontrolled hypertension (12% vs. 0.04%; OR [95%CI]; 1.88 [1.63, 2.16]). Similar results were also observed when OSA severity was categorized, and in models further adjusted for total sleep time (Supplementary Table 2). In the primary snoring analysis, further adjusting for total sleep time also did not change the main findings, and snoring duration in this model was associated with a 73% increase in uncontrolled hypertension (OR [95%CI]; 1.73 [1.38, 2.18]).

After excluding blood pressure entries not taken during the morning, 5383 (43.7%) participants remained for the sensitivity analysis. Of these, 1170 (21.7%) cases of hypertension were observed. In this model, snoring duration was associated with a 95% increase in uncontrolled hypertension (13% vs. 0.06%; OR [95%CI]; 1.95 [1.59, 2.40]). The main results regarding snoring and OSA severity category (Supplementary Table 2) and the results in primary snorers (data not shown), also remained the same.

## Discussion

This study indicates that 15% of the studied population, which mostly comprised overweight men, snore on average for more than 20% of the night and that ~10% of participants without sleep apnea snore more than 12% of the night. The current findings also demonstrate that regular nightly snoring is associated with elevated blood pressure and uncontrolled hypertension, independent of OSA presence or severity. These findings provide important insight into the potential consequences of snoring on hypertension risk and highlight the need to consider snoring as part of clinical care and management of sleep problems, particularly in the context of hypertension management.

There is considerable variability in night-to-night measures of OSA severity^18–20^. Thus, repeated measures over multiple months, as performed in the current investigation, provides substantially greater precision around estimates of OSA severity and snoring than previous studies that have relied on single night recordings^22^. Moreover, the large-scale dataset surpasses all previous studies that have sought to investigate potential relationships between snoring and hypertension. Objective data were also collected in the naturalistic home environment using simple, low-cost consumer devices with established validation data. Multi-night measures in the home setting are more directly relevant to real-world risk exposure compared to data collected in sleep laboratory settings. Indeed, laboratory testing is typically derived from a single night in an unfamiliar environment, which may confound both sleep and blood pressure measures.

Nonetheless, there are several limitations of note. Firstly, there was a lack of assessment for clinical covariates that may confound the association between snoring duration and blood pressure, such as diet, exercise, alcohol, caffeine, tobacco use, and medications^23,24^. For example, smoking and alcohol consumption are associated with greater incidence and intensity of snoring and may have influenced the magnitude of the detected association between snoring and hypertension^25,26^. There was also no information on potential OSA treatment, such as continuous positive airway pressure or mandibular advancement therapy. Furthermore, estimated AHI from the under-mattress sensor includes fewer input variables from which detect respiratory events compared to conventional polysomnography. Although the estimated AHI by the WSA has good agreement with polysomnography^18,27,28^, the interdependence with snoring, one of the key inputs, may have influenced, at least to some extent, the detected association between snoring and blood pressure. The relative contribution of central vs. obstructive events to the prevalence of snoring and to the interaction between OSA severity and snoring in the association with hypertension also remains unclear and warrants further investigation. Validation of the device-estimated total sleep time also suggests that the device overestimate total sleep time by ~30 mins on average^29^, similar to other sleep wearables^30,31^. Given that snoring was expressed as a % of total sleep time in our study, this may have led to an under-estimation of the association between of snoring on blood pressure.

Participants were also predominantly male, which limits generalization and raises the need for caution when interpreting sex difference comparisons. Participants were also self-selected via their decision to purchase and regularly use the under-the-mattress sleep sensor and blood pressure monitor devices from which these data were derived. Consumer choices could reflect concerns about sleep and blood pressure, potentially contributing to a bias towards overestimating snoring prevalence. On the other hand, consumer engagement could potentially be biased towards those who are more engaged in their own health. The data was also collected during the COVID-19 pandemic, and COVID-19 infections may have influenced some of the observed effects.

Without any agreed standardized approaches as to how snoring should be assessed, meaningful comparisons of snoring prevalence across studies are problematic. For example, in our study, ~15% of the population snored on average more than 20% of their estimated sleep time. In the Busselton study, 22% of the studied population snored more than 50% of the total sleep time^4^. Notwithstanding this limitation, the estimated snoring prevalence across OSA severity category is similar to the literature^5,32^. Standardized algorithms and methods for snoring assessment are clearly needed for meaningful comparisons of snoring prevalence across studies. Importantly, snoring algorithm design and cut-off choices should be informed by the associated adverse health impacts of snoring, such as hypertension as reported in the current investigation.

It is also unclear how effectively the under-the-mattress sensors detect snoring from the participant and not a bed partner. This may have led to imprecise detection of snoring and influenced the estimated risk for uncontrolled hypertension associated with snoring. Finally, the under-the-mattress sleep sensor does not evaluate snoring intensity, which was shown to be associated with increased next-morning blood pressure^8^. Thus, the evaluation of snoring loudness is also likely to be beneficial. Similarly, recent evidence suggests that snoring acoustic characteristics may be useful markers to detect the site(s) of upper airway collapse in people with OSA^33^. Analysis of snoring acoustic characteristics and intensity from under-the-mattress sensors, or potentially bed-side devices, therefore, warrants further investigation in OSA and in snorers.

Identifying associations between blood pressure and uncontrolled hypertension with objective, multi-night snoring recordings is consistent with previous much smaller studies and those reliant on subjective snoring assessments^8,9,34^. The moderating effect of age and BMI on the associations between snoring duration and hypertension is also consistent with prior OSA literature. Previous studies suggest that downstream effects of OSA per se on adverse health outcomes may be stronger in younger participants and potentially influenced by protective hypoxic pre-conditioning effects in older people with OSA^34,35^. Bixler and colleagues^35^ found that the association between OSA severity (measured using the AHI) and hypertension was stronger in younger and non-obese participants. Similarly, we found moderating effects of age and BMI, whereby snoring duration was more strongly associated with hypertension in younger and non-obese participants. This group, which does not present with the usual co-morbidities associated with snoring and OSA, may therefore be a sensible group to target with therapeutic interventions in future studies to reduce snoring and potentially blood pressure.

We have previously shown that AHI and inter-night variability of AHI estimated using the same under-the-mattress device are independently associated with hypertension risk^36^. Another recent study has shown that increased respiratory effort detected by mandibular jaw movement, some of which likely translated into snoring, measured using a device placed on the chin was independently and strongly associated with prevalent hypertension in 1127 patients^37^. Together, these findings indicate that non-invasive in-home monitoring over multi-nights using novel technologies may be clinically useful to better detect snoring and OSA, their potential adverse cardiovascular consequences, and to inform therapeutic decision making including in primary care.

In our study, the association between severe OSA-alone and blood pressure had a similar effect size to the association between snoring-alone and blood pressure. This finding supports that snoring may be an important mechanism that contributes to hypertension. Furthermore, the observed effect sizes were similar to prior literature. For example, in the Sleep Heart Health Study, severe OSA (AHI > 30) vs. no-OSA (AHI < 1.5 events/h) was associated with a 3.4 mmHg increase in diastolic blood pressure, independent of age, sex, and BMI^23^. In this study, regular nightly snoring was associated with up to 4.5 mmHg higher diastolic blood pressure after adjustment for age, sex, BMI, and OSA severity. While uncontrolled factors may explain some of this effect (e.g., smoking or alcohol), snoring could also represent an independent mechanistic pathway via which chronically obstructed breathing during sleep contributes to elevated blood pressure. For example, via intrathoracic pressure effects on baroreceptor mediated control of systemic (and potentially pulmonary) blood pressure or potentially via carotid baroreceptor effects from mechanical vibration emanating from the pharyngeal airway^8,9,11–13^. Observational epidemiological studies have shown a strong and linear association between blood pressure and the risk of cardiovascular disease and cardiovascular death without any evidence of a blood pressure threshold^24^. The Prospective Studies Collaboration^38^ estimated that an increase in systolic blood pressure of 20 mmHg, or diastolic blood pressure of 10 mmHg, was associated with a more than twofold increase in the rate of cardiovascular death. Therefore, the increase in blood pressure associated with sleep apnoea, snoring, and their combination (~6 mmHg increases in systolic and diastolic blood pressure compared to no snoring or OSA) has important clinical relevance.

The elevated hypertension prevalence in people with primary snoring (eAHI< 5 events/h) was comparable to other OSA severity categories. This finding further suggests that hypertension risks associated with snoring may be independent of OSA and potentially more strongly related to chronically obstructed breathing rather than blood gas or sleep disturbance effects. These results are concordant with previous studies where associations between snoring loudness and elevated morning blood pressure in patients with an eAHI< 15 events/h were detected^8,9^. Low-level continuous positive airway pressure has been shown to significantly reduce snoring in participants without OSA^39^. Whether a reduction in snoring is associated with a decrease in blood pressure and other cardiovascular risk factors clearly warrants further investigation.

In summary, long-term nightly snoring assessments indicate that snoring is highly prevalent in the adult community and is associated with a ~20 to 80% increase in hypertension prevalence, independent of OSA severity. High hypertension prevalence was also observed for people with a high proportion of the night spent snoring, even without sleep apnea (eAHI<5 events/h). These findings provide important insight into the potential consequences of snoring to hypertension. Thus, further investigation is warranted to determine whether therapeutic interventions directed toward snoring can reduce hypertension, one of the leading risk factors for cardiovascular disease and mortality.

## Methods

### Participants

This study analyzed data from 12,287 participants who registered to use both an under-the-mattress sleep sensor (Withings Sleep Analyzer: WSA) and an FDA-registered home-blood pressure monitor between July 2020 and April 2021. Further inclusion criteria were ≥28 nights of sleep and snoring recordings and at least five separate blood pressure measurements over the recording period for assessment of uncontrolled hypertension outcomes. All participants provided written consent through the Withings app for their deidentified data to be used for research purposes when signing up for a Withings account, and the current study was approved by the Flinders University Human Research Ethics Committee (Project number: 4291).

### Snoring and blood pressure assessments

The WSA is a nonwearable sleep monitoring device placed under-the-mattress that detects snoring and estimates the apnea-hypopnea index (estimated AHI; eAHI) and sleep stages. This is achieved via automated proprietary algorithms from a built in microphone and ballistographic assessment of movement, heart rate, and respiratory motion from a pressure sensor^28^. The estimated AHI has good agreement with in-laboratory polysomnography-derived AHI with high predictive performance to classify mild (89% sensitivity and 75% specificity), moderate-to-severe (88% sensitivity and 88% specificity), and severe OSA (86% sensitivity and 91% specificity)^18,27,28^. The estimated AHI also has minimal bias with in-laboratory polysomnography derived AHI when the AHI is considered as a continuous variable^28^. Features characteristic of the power spectrum of snoring sound are calculated, and snoring is scored over 1-minute epochs. To be classified as a snoring epoch, ≥50% of the epoch must contain snoring. A post-processing window over 3 consecutive 1 min epochs is then applied to define snoring. This has the advantage of limiting the number of false positives due to extraneous noise. Snoring duration is then expressed as a percentage of total sleep time by dividing snoring time by the estimated total sleep time (hereafter termed “snoring duration”).

Blood pressure values were obtained via Withings blood pressure monitor measurements undertaken by each participant. The home-blood pressure monitor comes with instructions that outline how to take a blood pressure measurement. Specifically, the user is instructed to 1) rest for ≥5 minutes before taking a measurement, 2) be seated in a comfortable position and in a quiet area with legs uncrossed, feet flat on the floor, and back/arm supported, 3) not speak during the measurements and 4) perform the measurement on their left arm. Uncontrolled hypertension was defined as a mean systolic blood pressure ≥140 mmHg or mean diastolic blood pressure ≥90 mmHg^40^ averaged across all available blood pressure measurements over the monitoring period.

### Statistical analysis

The primary exposure variables were mean eAHI and mean snoring duration across all available nights of data. OSA severity categories were defined using standard clinical cut-offs^3^ (<5 events/h sleep = no OSA, $\ge$5 and <15 = mild, $\ge$15 and <30 = moderate and ≥30 events/h sleep = severe OSA). Snoring was categorized using quartiles and tertiles where necessary for interpretation but was otherwise kept as a continuous variable in the analyses.

The prevalence of snoring and mean snoring duration were examined separately by sex. Linear regression models controlling for age, sex, BMI, and mean eAHI were used to investigate the potential association between snoring duration and systolic and diastolic blood pressure. Odds ratio (ORs) and 95% confidence interval (CIs) were determined using logistic regression models to assess the association between mean snoring duration and uncontrolled hypertension adjusted for age, sex, BMI, and average eAHI.

eAHI and mean snoring duration were modelled using restricted cubic splines to account for potential non-linear associations. Interactions between snoring duration with age, sex and BMI were also investigated. When interactions were significant, BMI was categorized as normal (<25 kg/m²), overweight (BMI between 25 and 30 kg/m²), and obese (BMI ≥ 30 kg/m²). When interactions with age were observed, age was categorized using a median split. As a secondary analysis, snoring duration was categorized using tertiles, and the contribution of snoring in each OSA category (from mean eAHI) was examined using logistic regression adjusted for available confounders. Logistic and linear regression were performed in the R programming language.

### Sensitivity analyses

To further validate our findings, we conducted two additional sensitivity analyses. In the first sensitivity analysis, given the association between total sleep time and hypertension^29^, we additionally adjusted our models for total sleep time. Given that blood pressure may vary during the day, we performed an additional sensitivity analysis where only morning blood pressure entries, measured between 6am and 12 pm, were included.

### Reporting summary

Further information on research design is available in the Nature Research Reporting Summary linked to this article.

### Supplementary information

Supplementary material formatted

Reporting Summary

## Data Availability

Deidentified data that support the findings of this study, including individual data, are available from the corresponding author upon request subject to ethical and data custodian (Withings) approval.

## Code availability

Code for the analysis is available upon reasonable request from the corresponding author.
