# Supplementary material for: Regular snoring is associated with uncontrolled hypertension
Source: NPJ Digit Med. 2024 Feb 17;7:38. doi: 10.1038/s41746-024-01026-7 (PMC10874387; doi:10.1038/s41746-024-01026-7)
Supplement: Supplementary file 1 — Supplementary material formatted [file 41746_2024_1026_MOESM1_ESM.docx]

**Supplementary material: Regular snoring is associated with uncontrolled hypertension**

Bastien Lechat^1*^, Ganesh Naik^1^, Sarah Appleton^1^, Jack Manners^1^, Hannah Scott^1^, Duc Phuc Nguyen^2^, Pierre Escourrou^3^, Robert Adams^1^, Peter Catcheside^1^, Danny J Eckert^1^

* corresponding author

[bastien.lechat@flinders.edu.au](mailto:bastien.lechat@flinders.edu.au)

Mark Oliphant Building, Level 2, Building A, 5 Laffer Drive, Bedford Park 5042

^1^ Adelaide Institute for Sleep Health and FHMRI Sleep Health, College of Medicine and Public Health, Flinders University, Adelaide, Australia

^2^ College of Science and Engineering, Flinders University, Adelaide, Australia

^3^ Centre Interdisciplinaire du Sommeil, Paris, France

## Supplementary notes

## Snoring and blood pressure

There was an interaction between snoring duration and age (p= 0.008) in the association between snoring and diastolic blood pressure, where the effect of snoring duration on diastolic blood pressure was more pronounced in younger participants (Figure 1). Furthermore, the association between snoring duration and diastolic blood pressure was higher (e.g., steeper increase; p-value for interactions, p <0.001) for participants with a BMI ≥ 30 compared to participants with a BMI < 30 kg/m².

Similarly, there was a significant interaction with BMI (p < 0.001) but not age (p = 0.600) in the association between snoring duration and systolic blood pressure. There was also an interaction between snoring duration and sex (Supplementary Table S1, p < 0.001) in the association with systolic blood pressure. Conversely, to diastolic blood pressure, participants with a BMI < 30 kg/m² (Figure 1) and women (Supplementary Table S1) had a higher increase in systolic blood pressure associated with snoring duration than obese participants and men.

## Supplementary Figures


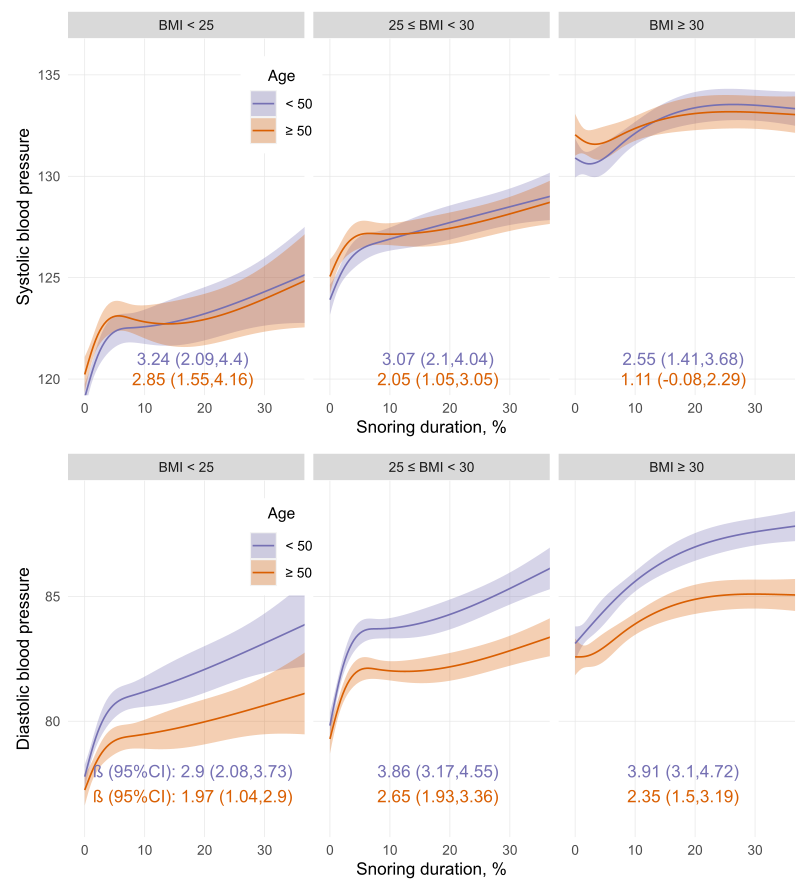


**Supplementary Figure 1.** *Associations between snoring duration with systolic and diastolic blood pressure, modelled using 3 knots restricted cubic spline for snoring duration and interaction with age categories in years (median split) and BMI categories (kg/m^2^). β (95%CI) represents the difference between the 5% and the 75% percent of the snoring duration distribution. Note that the 5% and 75% percent were determined separately for each BMI category.*

*
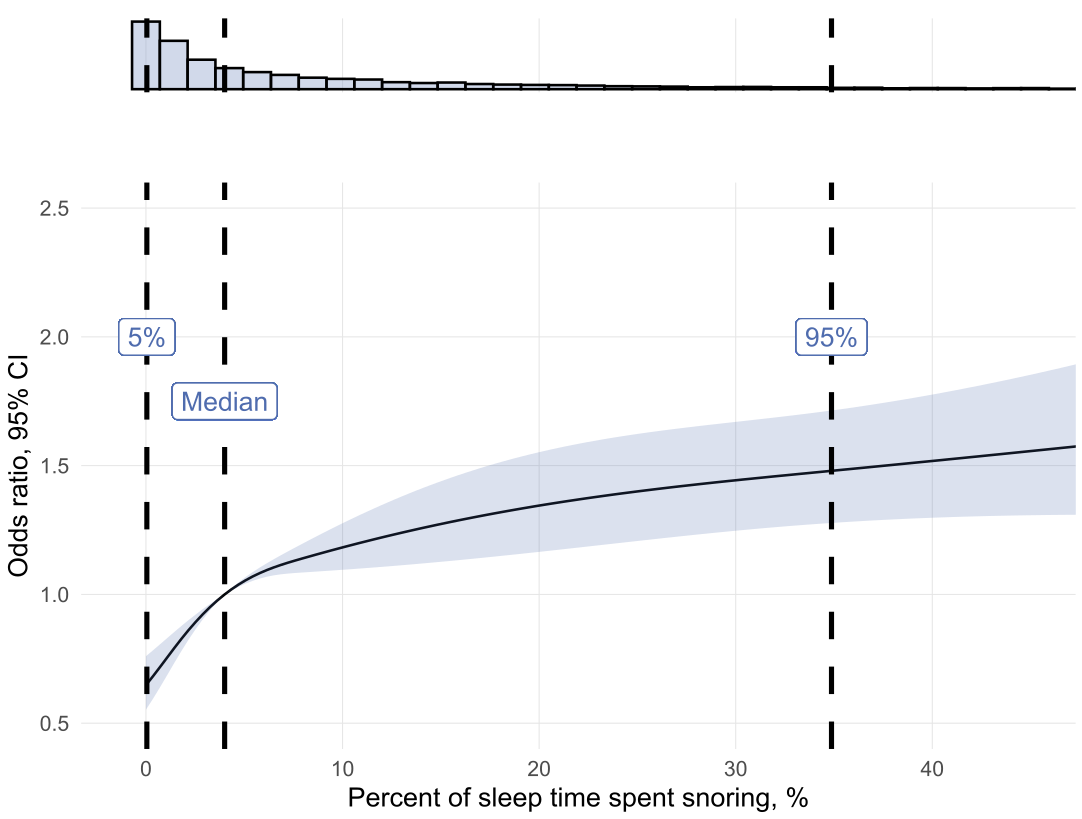
*

***Supplementary Figure 2.*** *Associations between snoring (as a % of total sleep time) and hypertension risk. Dashed lines represents 5^th^, 50^th^ and 95^th^ percentiles of the distribution to show the spread of the data.*

*
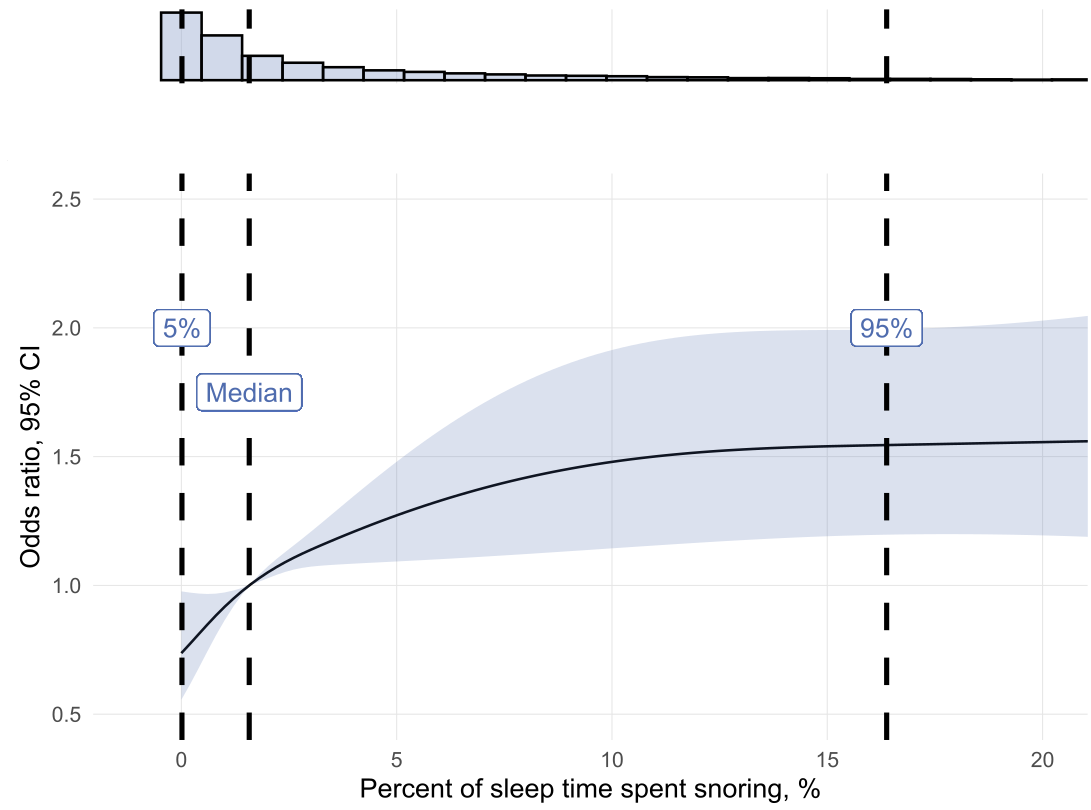
*

***Supplementary Figure 3.*** *Associations between snoring (as a % of total sleep time) and hypertension risk in participants with no sleep apnoea (apnoea-hypopnea index < 5). Dashed lines represents 5^th^, 50^th^ and 95^th^ percentiles of the distribution to show the spread of the data.*

## Supplementary Tables

***Supplementary Table 1****: Association between snoring and systolic blood pressure modelled with an interaction term between snoring and BMI categories and an interaction term between snoring and sex. Results represent the blood pressure difference β (95%CI) between the 75^th^ vs. 5^th^ percentile of the snoring distribution. Models are adjusted for age and apnea-hypopnea-index.*

|  | Men | Women |
| --- | --- | --- |
| BMI < 25 | 2.71 (1.61, 3.81) | 3.90 (2.06, 5.74) |
| 25 < BMI < 30 | 2.17 (1.34, 3.00) | 5.20 (3.53, 6.87) |
| BMI > 30 | 1.47 (0.44, 2.50) | 5.33 (3.50, 7.15) |

***Supplementary Table 2.*** *Associations between hypertension likelihood with obstructive sleep apnea (OSA) severity categories and quartile of % of sleep time spent snoring.*

*Displayed values are ORs (95% CI) of the association between an OSA/snoring group with the reference group (no-OSA and lowest snoring quartile). Models were adjusted for sex, age ,BMI, and total sleep time.*

|  |  | **Quartiles of percent of sleep time spent snoring** | | | |
| --- | --- | --- | --- | --- | --- |
|  |  | **0 to 0.9%** | **0.9 to 4%** | **4 to 12%** | **12 to 83%** |
| **Obstructive sleep apnea severity**  **category^1^** | **No OSA** | 1 (ref) | 1.47  (1.17, 1.86) | 1.81  (1.42, 2.30) | 2.73  (2.05, 3.64) |
|  | **Mild** | 1.50  (1.16, 1.94) | 1.86  (1.47, 2.37) | 2.32  (1.85, 2.90) | 2.86  (2.27, 3.58) |
|  | **Moderate** | 1.57  (1.09, 2.27) | 2.02  (1.49, 2.76) | 2.32  (1.77, 3.03) | 3.08  (2.45, 3.89) |
|  | **Severe** | 2.56  (1.59, 4.14) | 3.04  (2.04, 4.52) | 2.82  (2.01, 3.96) | 3.94  (3.08, 5.03) |

***Supplementary Table 3.*** *Associations between hypertension likelihood with obstructive sleep apnea (OSA) severity categories and quartile of % of sleep time spent snoring.*

*Only morning blood pressure entries are considered in this model. Displayed values are ORs (95% CI) of the association between an OSA/snoring group with the reference group (no-OSA and lowest snoring quartile). Models were adjusted for sex, age ,BMI, and total sleep time.*

|  |  | **Quartiles of percent of sleep time spent snoring** | | | |
| --- | --- | --- | --- | --- | --- |
|  |  | **0 to 1%** | **1 to 4.7%** | **4.7 to 13%** | **13 to 72%** |
| **Obstructive sleep apnea severity**  **category^1^** | **No OSA** | 1 (ref) | 1.57  (1.10, 2.22) | 2.36  (1.65, 3.37) | 2.88  (1.84, 4.50) |
|  | **Mild** | 1.79  (1.24, 2.60) | 1.79  (1.26, 2.54) | 2.45  (1.76, 3.41) | 2.99  (2.13, 4.20) |
|  | **Moderate** | 1.85  (1.12, 3.07) | 2.23  (1.44, 3.44) | 2.69  (1.82, 3.96) | 3.40  (2.41, 4.77) |
|  | **Severe** | 1.70  (0.78, 3.72) | 2.94  (1.62, 5.34) | 2.49  (1.54, 4.03) | 4.33  (3.02, 6.21) |
